# Boosting the performance of pretrained CNN architecture on dermoscopic pigmented skin lesion classification

**DOI:** 10.1111/srt.13505

**Published:** 2023-10-24

**Authors:** Erwin Setyo Nugroho, Igi Ardiyanto, Hanung Adi Nugroho

**Affiliations:** ^1^ Engineering Faculty, Department of Electrical Engineering and Information Technology Universitas Gadjah Mada Yogyakarta Indonesia; ^2^ Department of Informatics Politeknik Caltex Riau Riau Indonesia

**Keywords:** augmentation, Bayesian tuning, convolutional neural network, hyper‐parameter optimization, pigmented skin lesion, skin cancer

## Abstract

**Background:**

Pigmented skin lesions (PSLs) pose medical and esthetic challenges for those affected. PSLs can cause skin cancers, particularly melanoma, which can be life‐threatening. Detecting and treating melanoma early can reduce mortality rates. Dermoscopic imaging offers a noninvasive and cost‐effective technique for examining PSLs. However, the lack of standardized colors, image capture settings, and artifacts makes accurate analysis challenging. Computer‐aided diagnosis (CAD) using deep learning models, such as convolutional neural networks (CNNs), has shown promise by automatically extracting features from medical images. Nevertheless, enhancing the CNN models' performance remains challenging, notably concerning sensitivity.

**Materials and methods:**

In this study, we aim to enhance the classification performance of selected pretrained CNNs. We use the 2019 ISIC dataset, which presents eight disease classes. To achieve this goal, two methods are applied: resolution of the dataset imbalance challenge through augmentation and optimization of the training hyperparameters via Bayesian tuning.

**Results:**

The performance improvement was observed for all tested pretrained CNNs. The Inception‐V3 model achieved the best performance compared to similar results, with an accuracy of 96.40% and an AUC of 0.98.

**Conclusion:**

According to the study, classification performance was significantly enhanced by augmentation and Bayesian hyperparameter tuning.

## 1 INTRODUCTION

Pigmented skin lesions (PSLs) can be medically and esthetically problematic, with melanoma being the most concerning form as it is a type of skin cancer. In the United States, it is estimated that one in five Americans will develop skin cancer by age 70, and more than two people die every hour from this condition. Having frequent sunburns increases the risk of developing melanoma. Although the number of newly diagnosed cases is predicted to decrease by 2023, the number of deaths from melanoma is expected to rise. However, early detection significantly increases the 5‐year survival rate for melanoma, which currently stands at 99%.^1^

Dermoscopic images and biopsy images are commonly used to examine PSLs. Dermoscopic images are preferred as they are noninvasive, quick, and cost‐effective.^2^, ^3^, ^4^ However, there are challenges in accurately interpreting these images, such as the presence of artefacts, variability within and between image classes, and subjectivity in reading by doctors.

In recent years, machine learning, particularly deep learning, has been used to aid in PSL classification. Deep learning has the advantage of being able to directly process raw data without the need for extensive pre‐processing methods. Deep learning enables computer‐aided diagnostic (CAD) to detect abnormalities and signs of disease with higher accuracy, even beating doctors.^5^, ^6^, ^7^ Current research on PSLs focuses on three main areas: segmentation,^8^, ^9^, ^10^, ^11^, ^12^ feature extraction,^13^, ^14^, ^15^, ^16^, ^17^ and classification.^18^, ^19^, ^20^, ^21^, ^22^, ^23^ Furthermore, the field of interpretable machine learning or explainable artificial intelligence is expanding to address ethical concerns in the healthcare industry.^24^

This research will use pretrained CNN for PSL classification with the ISIC‐2019 dataset. The main contribution of this research is using augmentation to overcome dataset imbalance and hyper‐parameter optimization to improve the model performance. With these two treatments, the pretrained CNN achieves satisfactory performance and exceeds the existing results of similar studies. In the following, we list the contributions of this study:

1. We added preprocessing steps to improve the classification performance, such as data normalization, resizing, and augmentation.
2. We applied hyper‐parameter optimization with Bayesian tuning on the added learning and dropout parameters.
3. We are implementing pretrained CNN with ImageNet transfer learning and adding a dropout layer before the last layer.

This paper is presented with a structure: Section 1 is related to the background, this research's importance, and contribution statement. Section 2 presents related works, followed by materials, methods, and experiment scenarios in Section 3. Section 4 discusses the results and discussion, and Section 6 concludes.

## 2 RELATED WORK

The related research described in this section explicitly classifies PSLs with the ISIC 2019 with eight classes dataset. Molina et al.^25^ used DenseNet‐201 with three classifiers to perform PSL classification with the ISIC‐ 2019 dataset. They performed augmentation to address class data imbalance. While this approach resulted in high accuracy and precision, it was less successful in improving sensitivity, an essential parameter in the medical field that should not be overlooked. Meanwhile, the augmentation process performed by Liu et al.^26^ did not describe the methods and procedures used, and the performance results obtained were also unsatisfactory.

Filipescu et al.^27^ employed a pretrained CNN VGG‐16 to classify PSLs in the ISIC‐2019 dataset, comprising eight classes. The researchers conducted preprocessing by resizing images to match the pre‐trained model and optimizing hyper‐parameters. The accuracy achieved was 78.11%.

Cauvery et al.^28^ addressed the problem of unbalanced classes by using an online augmentation policy. Although this method has the indirect advantage of increasing the number of training images, it has several disadvantages, including dependence on online connection, higher computational cost, dependence on input data quality, and risk of over‐fitting. Like the work of Kassem et al.,^29^ our study also incorporates the concept of augmentation, where the number of images in each class is augmented to approximate the number of images in the largest class.

## 3 MATERIAL AND METHODS

This section describes the CNN from this study that was used for the classification of PSLs. This study identifies the impact of the pretrained CNN model on the ISIC 2019 dataset when applying augmentation and Bayesian tuning.

### 3.1 Dataset

The dataset used in this study is the 2019 ISIC dataset.^34^, ^35^, ^36^ This dataset was used for the 2019 International Skin Imaging Collaboration competition. This dataset was chosen because it has many images and the most classes at this time. The data contain dermoscopy‐captured images and metadata. ISIC 2019 dataset comprises eight disease classes with imbalance distribution, almost half melanocytic nevus (NV).

Data preprocessing steps such as duplicate detection, data cleaning, and resizing were initially performed. Duplicate detection ensured that no subset of the test data was present in the training data subset. Data cleaning involved removing images without lesion IDs in their metadata and resizing adjusted the size of input images to match the required dimensions of the CNN model. Following preprocessing, 800 images, with 100 images per class, were set aside for testing. In contrast, the remaining images were used for training data after undergoing augmentation to balance the data distribution.

The augmentation was carried out using the Image Data Generator with the following settings: rotation (180º), height and width shifting (0.1), zooming (0.1), horizontal and vertical flipping, brightness adjustment (ranging from 0.9 to 1.1), and fill mode (nearest) to increase the diversity and variability of the training data. This process utilized a batch size of 20 and aimed to generate 9200 final images, corresponding to the largest number of images in the NV class. The results of the preprocessing and augmentation are summarized in Table 1.

**TABLE 1:** Preprocessing, splitting and augmentation ISIC‐2019 dataset.

Class	Initial data	No ID lesion	Testing dataset	Training dataset	Augmented training dataset
(1) Melanocytic nevus (NV)	12 875	3647	100	9128	9128
(2) Melanoma (MEL)	4522	495	100	3927	9204
(3) Basal cell carcinoma (BCC)	3323	138	100	3085	9220
(4) Benign keratosis (BKL)	2624	436	100	2088	9202
(5) Actinic keratosis (AK)	867	36	100	731	9060
(6) Squamous cell carcinoma (SCC)	628	24	100	504	8606
(7) Dermatofibroma (DF)	239	11	100	128	8436
(8) Vascular lesion (VASC)	253	39	100	114	7918
Total	25 331	4826	800	19 705	70 774

### 3.2 CNN models

Convolutional neural network (CNN) is a type of deep learning consisting of convolution layers as feature extractors and fully connected neural layers as classifiers. Information is formed or extracted by the convolution block and then fed to the fully connected block to generate predictions from the classification. Pretrained CNN is a model that has been trained with a specialized dataset. For the case of image classification, pretrained CNNs have usually been trained with the ImageNet Large Scale Visual Recognition Challenge (ILSVRC) or commonly called ImageNet (Russakovsky et al., 2015). ImageNet contains 1000 classes with 1 281 167 training images, 50 000 validation, and 100 000 test images. A pretrained CNN model is intelligent when trained with ImageNet and produces accuracy above 70% for top‐one accuracy and above 90% for top‐five accuracy. Currently, there are many pretrained CNN developed. Selected four models pretrained CNN models in this research as presented in Table 2. Consideration of the selection of CNN pretrained models is based on the following:

1. Models with parameters below 25 million parameters due to available resources.
2. Models with accuracy above 75% for top‐one accuracy and above 90% for top‐five accuracy.
3. Models with the last variant in the family.
4. Available in Keras.

**TABLE 2:** Description and general characteristic selected pretrained CNN.

Pretrained CNN	Top‐one accuracy (%)	Top‐five accuracy (%)	Depth	Parameter (million)	Input size
Inception‐v3^30^	77.9	93.7	189	23.9	299 × 299
DenseNet‐201 ^31^	77.3	93.6	402	20.2	299 × 299
Xception^32^	79.0	94.5	81	22.9	299 × 299
MobileNet‐v2^33^	76.2	93.2	105	3.5	224 × 224

### 3.3 Experiment scenario

**FIGURE 1:**
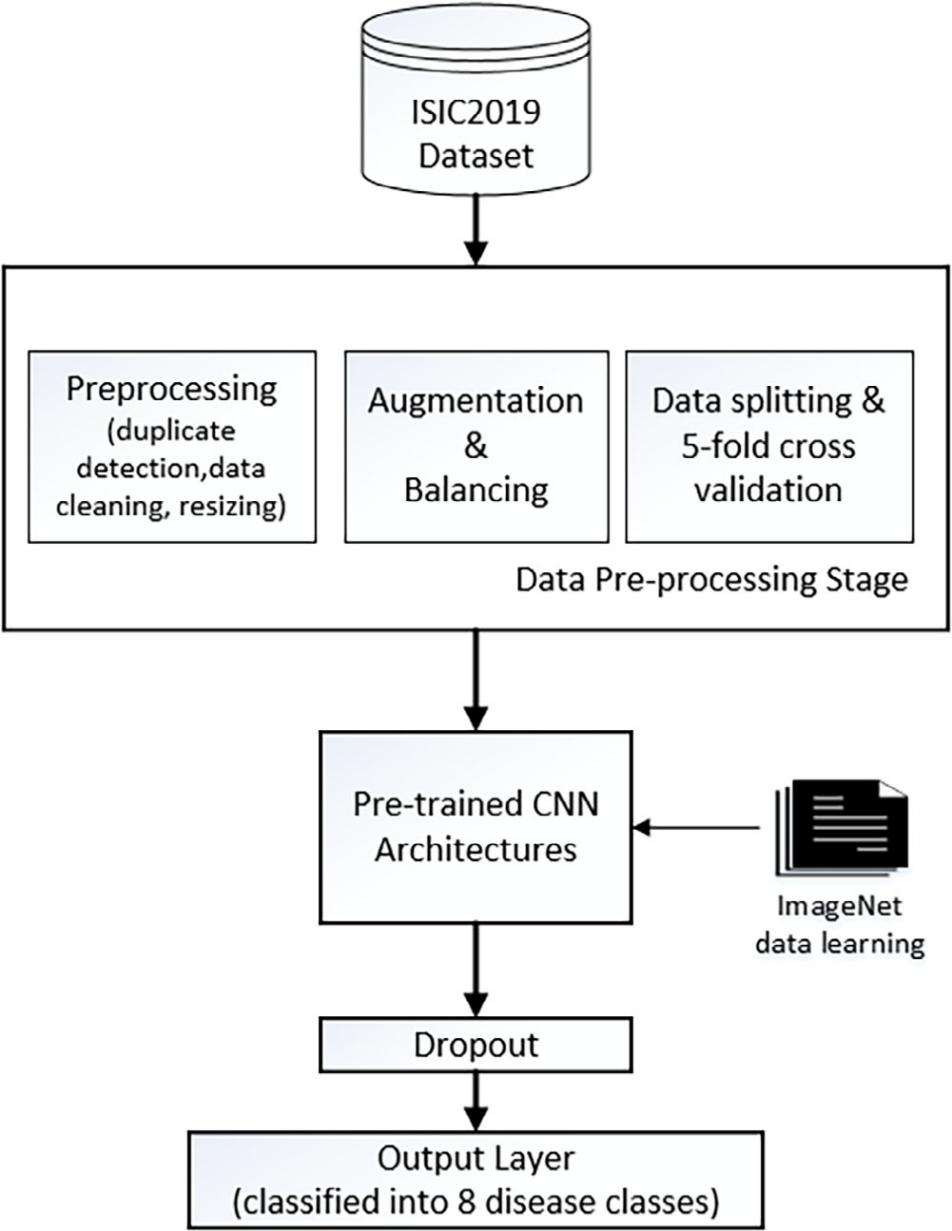
Experiment scenarios.

The experiments followed the flow chart shown in Figure 1. The ISIC‐2019 dataset of 15 331 dermoscopic images in eight classes of abnormality categories was taken as input. Preprocessing was performed in the form of duplication removal, image size adjustment, and separation of 100 images per class for image size adjustment and splitting of 100 images per class for testing data. The remaining data for training were previously augmented and aligned to the number of images per class.

Training process by applying five‐fold cross‐validation. The initial parameter configuration of the pretrained CNN was made the same as ADAM as the optimization algorithm, learning rate of 0, 01, dropout of 0, 1, batch size of 20 and epoch of 50 times. Parameter configuration is also done with Bayesian tuning for learning rate and dropout values. The results of tuning the defense rate and dropout values for each and dropout for each model are presented in Table 3.

**TABLE 3:** Result of Bayesian hyper‐parameter tuning.

Pre‐trained CNN model	Dropout	Learning rate	Optimizer
Inception‐v3	0.30	0.0001	ADAM
DenseNet‐201	0.20	0.0001	ADAM
Xception	0.40	0.0001	ADAM
MobileNet‐v2	0.20	0.0001	ADAM

The process runs on a device with 3.7‐GHz Intel Core i9‐ 10900K specifications, 128 GB RAM, and NVIDIA GPU. 128 GB RAM, 11 GB NVIDIA RTX 3080 GPU with Linux Ubuntu 18.04 and running JupyterHub application. Experiments will be conducted in three different treatments, namely:

1. the model without data augmentation,
2. the model with augmentation data,
3. the model with data augmentation and Bayesian tuning.

Algorithm 1 presents the Bayesian tuning procedure that will be implemented in this study. The input variables required in this procedure are the dataset *D*, the number of percentage subsets of the dataset *p*, the number of initial epochs $E_{\text{init}}$, the number of trials *n*, and the number of stopping epochs $E_{\text{stop}}$. This research uses 25% of the augmented dataset as training data for Bayesian tuning with the number of trials *n* = 5 and the objective function validation accuracy.

ALGORITHM 1 **Bayesian tuning (*D*, *p*, $E_{\text{init}}$, *n*, $E_{\text{stop}}$, *T*)**

**Table:** 

Define $D_{\mathrm{subset}}\leftarrow \mathrm{Sample}\left(D,p\right)$
Define $D_{\mathrm{train}},D_{\mathrm{val}}\leftarrow \mathrm{Split}\left(D_{\mathrm{subset}}\right)$
Define search space for hyperparameters θ
Define objective function f(θ) using validation accuracy
Initialize $E\leftarrow E_{\mathrm{init}}$, T←0
**while** T<n **do**
Train model with hyperparameters θ for *E* epochs
Implement early stopping based on
Update hyperparameters θ using Bayesian optimization
Update $E_{\mathrm{opt}}$ using the best validation accuracy so far
T←T+1
**end while**
Train with optimal hyperparameters $\theta^{*}$ for $E_{\mathrm{opt}}$ epochs
Save the model with the best hyperparameters $\theta^{*}$

### 3.4 Evaluation

Analyzing a learning algorithm on test data determines the algorithm's quality. The design of the evaluation matrix begins with the confusion matrix. The performance evaluation matrices commonly used in classification are sensitivity (SEN), specificity (SPE), accuracy (ACC), precision (PREC), and area under curve (AUC).

## 4 RESULT AND DISCUSSION

CNNs present a significant opportunity to address classification problems related to PSLs. The present study evaluated several pretrained models' ability to classify PSL diseases. In this work, the ISIC 2019 dataset with eight classification classes was used as input for four CNN architectures, namely Inception‐V3, DenseNet‐201, Xception, and MobileNet‐v2. The study evaluates the performance of these models.

### 4.1 Training models

**FIGURE 2:**
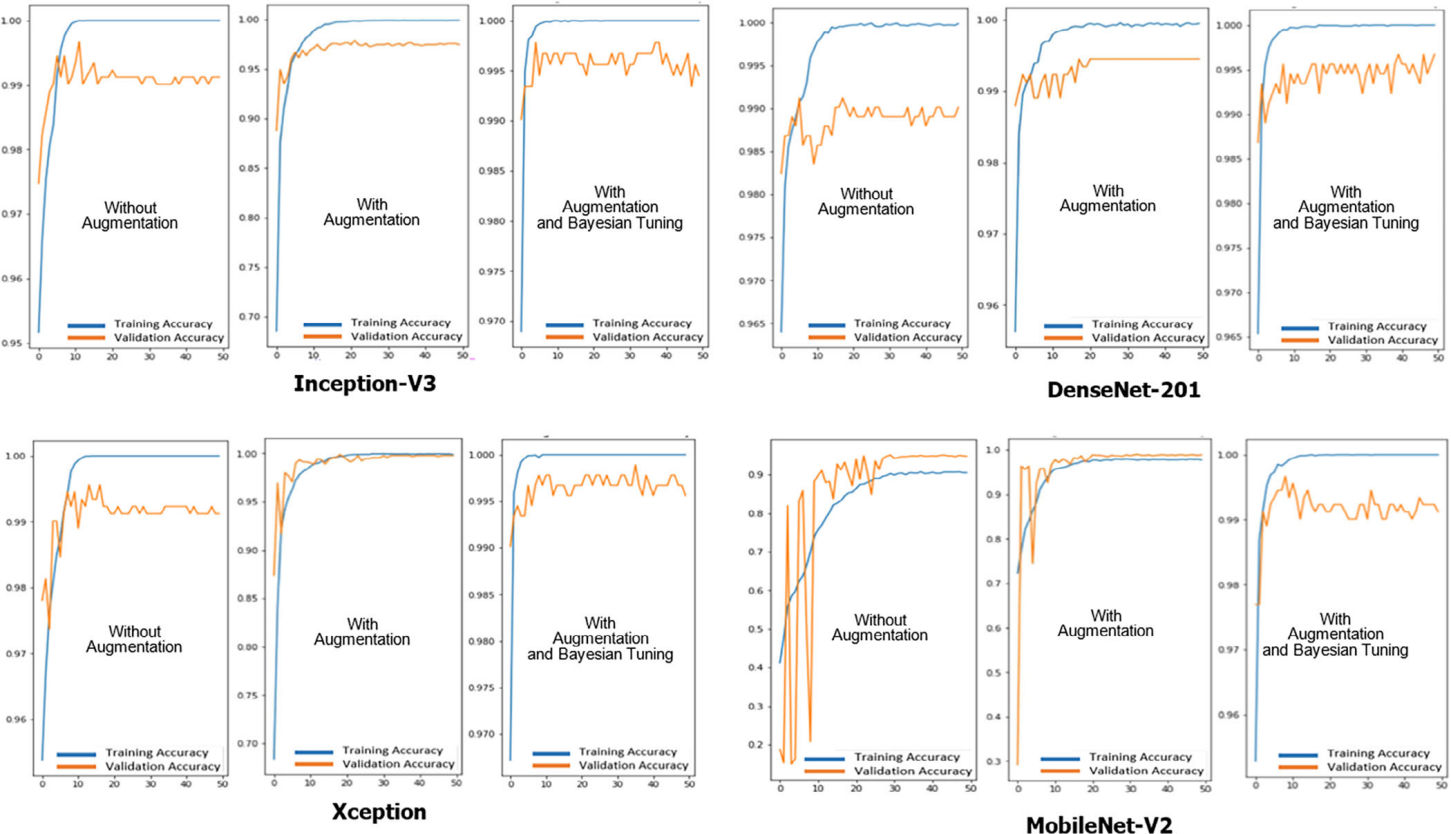
Training and validation accuracy of four models. The vertical axis depicts the accuracy value, while the horizontal axis depicts the number of epochs. A good model results from the validation accuracy pattern that follows the training accuracy pattern.

This stage ascertains whether a model is overfitting, underfitting, or fitting well. Models or architectures that fit well will achieve good test results. Figure 2 shows the training and validation accuracy for four models: Inception‐V3, DenseNet‐201, Xception, and MobileNet‐V2. The models were trained with three different treatments: no augmentation, with augmentation, and with augmentation along with Bayesian tuning. A good model results from the validation accuracy pattern following the training accuracy pattern. Here is our analysis of the graph:

- Inception‐V3 has the highest training accuracy, but its validation accuracy plateaus after 10 epochs. This indicates that the model is no overfit the training data.
- DenseNet‐201 has a lower training accuracy than Inception‐V3, but its validation accuracy continues to increase after 10 epochs. This indicates that the model does not overfit the training data.
- Xception has a similar training accuracy to DenseNet‐201, but its validation accuracy begins to decline after 15 epochs. This indicates that the model begins to overfit.
- MobileNet‐V2 has the lowest training accuracy of the four models, but its validation accuracy continues to increase throughout the training process. This indicates that the model is not overfitting the training data.

All four models demonstrate satisfactory performance during training. Overfitting remains minimal, particularly for models that are augmented and tuned using Bayesian methods. The model's performance in the testing phase will confirm this conclusion. Here are some additional observations about the graph:

- The models that were trained with augmentation performed better than the models that were not trained with augmentation. This suggests that augmentation can help prevent overfitting.
- The models that were trained with Bayesian tuning performed better than the models that were not trained with Bayesian tuning. This suggests that Bayesian tuning can help improve a model's generalization performance.

### 4.2 Testing models

**FIGURE 3:**
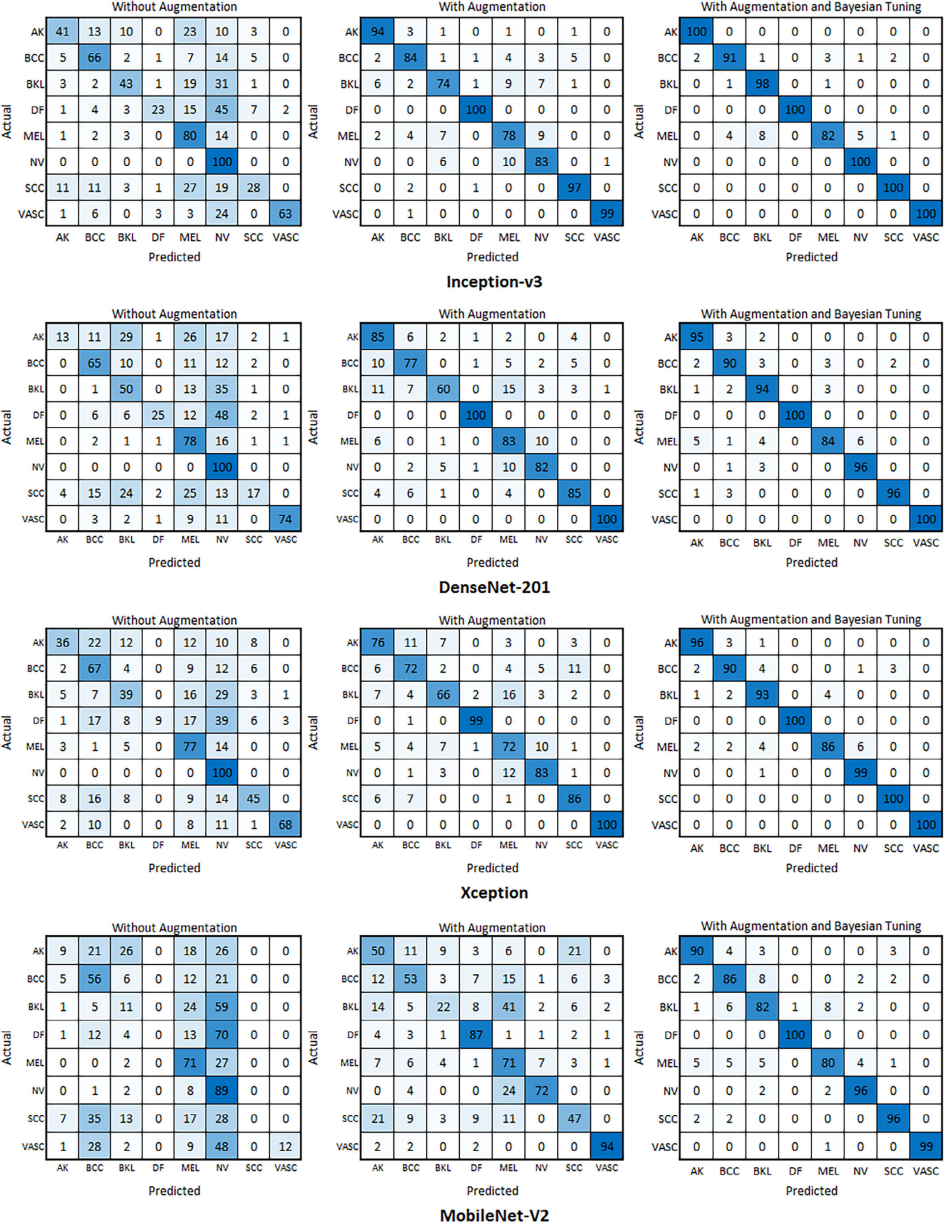
Confusion matrix testing of pretrained CNN for no augmentation, with augmentation, and with augmentation along with Bayesian tuning.

Once the training model has been completed, additional testing is conducted using data for testing purposes. The test data comprises images that are selected from the beginning of each class, with an interval of 100 images. The sensitivity, specificity, precision, accuracy, F1 Score, and AUC values for each model are calculated from the raw data contained in the confusion matrix. The confusion matrices of four CNN models for PSL classification with three treatments are presented in Figure 3.

Three conditions, namely without augmentation, with augmentation, and with augmentation as well as Bayesian tuning were used to train the models. The confusion matrices reveal the following observations:

- In general, better predictions were achieved with the models trained using augmentation and Bayesian tuning. As seen from the augmented and Bayesian tuning confusion matrices, the models exhibited more accurate predictions and fewer incorrect predictions.
- The Inception‐V3 model, with augmentation and Bayesian tuning, accurately detected actinic disease (AK), dermatofibroma (DF), melanocytic nevus (NV), squamous cell carcinoma (SCC), and vascular lesion (VASC).
- Melanocytic nevus (NV) can be accurately detected in models without augmentation, as the NV class has dominant data, constituting more than 50% of the training data.
- The accuracy distribution in models with augmentation and Bayesian tuning is even because both techniques balance training data between classes and optimize the model.

On the whole, the outcomes demonstrate that all four CNN models can accurately classify PSLs. Nevertheless, Inception‐V3 and DenseNet‐201 achieve a slightly superior performance when compared to Xception and MobileNet‐V2. Besides, augmenting the data and utilizing Bayesian optimization can enhance the performance of the models. Additional insights regarding the results are provided below:

- The findings indicate that augmentation is a crucial technique to enhance the performance of CNN models for the classification of skin lesions. This is due to the fact that augmentation mitigates overfitting and enhances the models' generalization ability.
- Another technique that can be applied to improve the performance of CNN models for skin lesion classification is Bayesian tuning. Bayesian tuning helps in identifying the optimal hyperparameters of the models resulting in improved accuracy.
- The results suggest that Inception‐V3 and DenseNet‐201 are better equipped to handle overfitting than Xception and MobileNet‐V2. This is probably because Inception‐V3 and DenseNet‐201 have deeper architectures.

Table 4 compares the performance of four pretrained CNN models under three treatments: no augmentation, with augmentation, and with augmentation along with Bayesian tuning. The metrics used to evaluate the models are Sensitivity (SEN), Specificity, Precision, Accuracy, F1 Score (F1), and Area Under the Curve (AUC). Here is a more detailed analysis of the results:

- Without augmentation:
  In this section of the table, the models' performance is evaluated without any data augmentation. The metrics suggest that the models' overall performance is relatively lower across the board. The highest Accuracy is around 55.50% for Inception‐v3, and the F1 Score also ranges between approximately 22.86 and 54.10%.
- With augmentation:
  When data augmentation is applied, the models' performance improves significantly across all metrics. This indicates that data augmentation helps the models to better generalize and perform well on new, unseen data. The Accuracy values are notably higher, ranging from 62.00 to 88.63%, with F1 Scores between approximately 61.19 and 88.53%.
- With augmentation and Bayesian tuning:
  The third section of the table introduces Bayesian tuning in addition to augmentation. Bayesian tuning is a hyperparameter optimization technique that can further enhance model performance. As expected, this combination leads to even better performance across all metrics. The Accuracy values are now in the range of 91.13%–96.38%, and F1 Scores are higher, ranging from around 91.08%–96.29%.

**TABLE 4:** Comparison of performance of four pretrained CNN models under three treatments: no augmentation, augmentation, and augmentation with Bayesian tuning.

Pretrained CNN model	Sensitivity (%)	Specificity (%)	Precision (%)	Accuracy (%)	F1 Score (%)	AUC
**Without augmentation**						
Inception‐v3	55.50	90.40	65.06	55.50	54.10	0.50
DenseNet‐201	52.75	89.41	64.06	52.75	49.29	0.50
Xception	55.13	64.97	64.71	55.13	52.22	0.50
MobileNet‐v2	31.00	80.07	31.88	31.00	22.86	0.50
**With augmentation**						
Inception‐v3	88.63	98.23	88.53	88.63	88.53	0.94
DenseNet‐201	84.00	97.40	84.60	84.00	83.86	0.90
Xception	81.75	96.98	81.78	81.75	81.69	0.89
MobileNet‐v2	62.00	92.27	63.29	62.00	61.19	0.78
**With augmentation and Bayesian tuning**						
Inception‐v3	96.38	99.47	96.40	96.38	96.29	0.98
DenseNet‐201	94.38	99.16	94.43	94.38	94.35	0.97
Xception	95.50	99.33	95.52	95.50	95.46	0.97
MobileNet‐v2	91.13	98.65	91.11	91.13	91.08	0.95

Here is a more detailed analysis of the results for each model:

- **Inception‐v3**: This model achieved the best performance on all metrics. Inception‐v3 is a relatively large model, which may explain why it performed so well. However, it is also a more computationally expensive model to train and deploy.
- **Xception**: This model achieved the second best performance on all metrics. Xception is a relatively new model that is designed to be efficient and accurate. It is a good choice for applications where both of these factors are important.
- **DenseNet‐201**: This model achieved the third best performance on all metrics. DenseNet‐201 is the deepest model among the four compared, which necessitates extensive computational resources.
- **MobileNet‐v2**: This model achieved the lowest performance on all metrics. However, it is also the smallest and most computationally efficient model of the four. MobileNet‐v2 is a good choice for applications where computational resources are very limited.

Table 4 demonstrates the clear advantages of using data augmentation and Bayesian tuning to enhance the performance of pretrained CNN models. These techniques help the models generalize better, resulting in higher accuracy and better overall performance in classification tasks.

Adding augmentation to the training process significantly improves the models' ability to generalize and make accurate predictions on new data. The combination of augmentation and Bayesian tuning further refines the models' performance, indicating that the models' hyperparameters are optimized to better fit the data. In terms of overall performance, Inception‐v3 consistently achieves the highest scores across all treatments, followed by DenseNet‐201, Xception, and then MobileNet‐v2.

Table 5 shows the duration of all models during training. The training time for all models decreases with data augmentation. This is surprising because data augmentation usually leads to a larger dataset, which consequently may result in longer training times. However, the augmented data may contribute to a faster convergence of the models due to increased diversity. When both data augmentation and Bayesian tuning are applied, the training time of Inception‐v3 and MobileNet‐v2 decreases significantly. This indicates that these models benefit from hyperparameter optimization and augmented data more than the other models. In all scenarios, Xception has the longest training times. This may be due to its architectural complexity, which may require more time for training convergence.

**TABLE 5:** Training duration of models.

	Training duration (min)		
Model	No augmentation	With augmentation	With augmentation and Bayesian tuning
Inception‐v3	427	178	98
DenseNet‐201	428	180	105
Xception	424	231	219
MobileNet‐v2	424	174	94

In summary, Table 5 emphasizes the trade‐off between training time and model performance optimization. Using techniques such as data augmentation and Bayesian tuning can potentially improve model performance, but they also increase training times.

### 4.3 Comparison with existing research

This section compares the results of this research with previous research. This comparison is limited to classification research using the eight‐class ISIC‐2019 dataset, as shown in Table 6. The performance of the four pretrained CNNs in this study with augmentation and Bayesian tuning can outperform almost all existing research results, except the accuracy performance is still defeated by Molina et al.^25^.

**TABLE 6:** Comparison of performance with existing research. Bolded values indicate the highest values.

Author	Method/Model	Performance matrix					
		Sensitivity (%)	Specificity (%)	Precision (%)	Accuracy (%)	F1 Score (%)	AUC
Molina et al.^25^	DenseNet‐201	66.45	97.85	91.61	**97.35**	n/a	n/a
Kassem et al.^29^	GoogleNet	79.80	97.00	80.36	94.92	80.07	n/a
Liu et al.^26^	Clinical‐Inspired	53.80	97.40	n/a	64.00	n/a	0.91
Cauvery et al.^28^	Ensemble CNN	62.00	98.00	73.00	81.00	56.00	n/a
Ours	Inception‐v3	**96.38**	**99.47**	**96.40**	96.38	**96.29**	**0.98**
Ours	Xception	94.38	99.16	94.43	94.38	94.35	0.97
Ours	DenseNet‐201	95.50	99.33	95.52	95.50	95.46	0.97
Ours	MobileNet‐v2	91.13	98.65	91.11	91.13	91.08	0.95

Molina et al.^25^ address the problem of unbalanced classes by using three classifiers with linear plurality voting. Although this approach achieves high accuracy and precision, it fails to improve sensitivity, a crucial parameter in the medical field that cannot be overlooked. Furthermore, this method requires extensive computational effort. While the augmentation process of Liu et al.^26^ lacks specific explanations regarding the methods and procedures used, the obtained performance results were also unsatisfactory.

Cauvery et al.^28^ tackle the problem of unbalanced classes by using an online augmentation policy. Although this method has the advantage of not directly increasing the number of training images, it has numerous drawbacks, including dependence on an online connection, higher computational cost, dependence on the quality of the input data, and the risk of overfitting. Like the research of Kassem et al.,^29^ our study incorporates augmentation concepts where the number of images in each class is increased to approach the number of images in the largest class. However, our research shows several advantages, especially with respect to the data cleaning and splitting processes. In particular, the test data are guaranteed to remain separate from the augmented training data. In Table 6, our model outperforms Kassem's model.

### 4.4 Limitations and future research

The limitation of this research is that it uses row images from the dataset and only performs augmentation and balancing between classes. It is possible to perform preprocessing to improve the quality of the input image so that the performance of the model can be improved.

For further research, it is still possible to improve the performance of the model by adding unique layers or modules such as attention, dense layers, and pooling or combining multiple models into an ensemble. Of course, this addition will increase the training time and requires optimization of appropriate parameters. To meet medical implementation requirements, it is necessary to conduct additional research for the model's interpretability.

## 5 CONCLUSIONS

The present study conducted an extensive evaluation concerning pretrained CNN models for the classification of PSLs, employing the ISIC 2019 dataset. The results highlighted the significance of data augmentation and Bayesian tuning techniques in enhancing the performance and generalizability of the models. Both Inception‐V3 and DenseNet‐201 consistently outperformed other models. This could be attributed to the effects of data augmentation and Bayesian tuning. Data augmentation helped prevent overfitting, and Bayesian tuning fine‐tuned the hyperparameters of the models.

Our models demonstrate promising outcomes, exceeding the accuracy and other metrics of various prior studies. However, we noted certain limitations, such as the use of raw images and the potential for further improvements through additional layers. Future research opportunities involve improving input quality through preprocessing and investigating advanced architectures. Our research establishes the foundation for accurate skin lesion classification using CNNs, which may provide potential for improved medical diagnosis and treatment.

## CONFLICT OF INTEREST STATEMENT

The authors declare no conflicts of interest.

## Data Availability

Dataset that supports the findings of this research (25,331 images) is openly available on ISIC‐2019 Challenge's website at https://challenge.isic‐archive.com/data/. The augmented image results and test data subsets are available from the corresponding author upon reasonable request.
